# The Nature of Consumption

**Published:** 1856-10

**Authors:** 


### HALL'S JOURNAL OF HEALTH.

OUR LEGITIMATE SCOPE IS ALMOST BOUNDLESS: FOR WHATEVER BEGETS PLEASURABLE AND HARMLESS FEELINGS, PROMOTES HEALTH; AND WHATEVER INDUCES DISAGREEABLE SENSATIONS, ENGENDERS DISEASE.

VOL. III.] OCTOBER, 1856. [NO. X.

### THE NATURE OF CONSUMPTION.

If a green bush is pulled up by the roots, and these roots are cut off close to the body of the bush, a good general idea may be had of the "*Air Passages*," if this bush is turned upside down. The end of the bush next the ground represents the part of the throat where the voice organs are, the body of the bush represents the windpipe, the branches of the bush represent the bronchial tubes, the leaves of the bush represent the lungs themselves; and as the leaves cover the branches from sight, so the lungs, which are nothing more than little bladders distended with air, hide the bronchial tubes. Here then are four distinct parts of a great apparatus, each different in locality, and each locality the subject of a distinct disease, requiring different remedies and a different treatment. Liquid guano destroys the leaf, but gives life to the roots. Water does but little good if thrown over a tree, but saves it from dying if thrown on the ground about it. So what would benefit one part of the air-passages, might be wholly unavailing if applied to another part. What would cure a disease of the windpipe, might destroy the lungs, or be perfectly useless. Thus showing how important it is to know certainly what the disease is, and where it is located, in reference to the great fountain of life, the breathing apparatus.

In the book called "*Bronchitis and Kindred Diseases*," 8th edition, these parallels are carried out minutely. Here it is sufficient to say, that when the disease is located at the voice organs, it is called *Throat-Ail* or chronic laryngitis. The common and well-known name of *Croup* is an affection of the windpipe. Bronchitis belongs to the branches of the windpipe; while consumption is a disease of the lungs themselves, destroying the little air bladders to which reference has been already made. Perhaps a diagram may illustrate more plainly.

| | | |
|---|---|---|
| Root, | Voice Organs, | Throat-Ail. |
| Body, | Windpipe, | Croup. |
| Branches, | Air Tubes, | Bronchitis. |
| Leaves, | Lungs, | Consumption. |

*Throat-Ail* gives a change of voice.

*Croup* gives difficult breathing.

*Bronchitis* gives a stuffed-up feeling.

*Consumption* gives steady emaciation.

Thus it is seen that throat-ail, or chronic laryngitis, is a disease at the top of the windpipe, where the voice organs are, its distinguishing feature being some change of the voice. Occasional additional feelings and symptoms are a huskiness of speech; sometimes the patient can only speak in the slightest whisper. Conversation is attended with an effort. Sometimes there is a painful feeling about the "swallow," a hurting sensation. At other times there is a pricking in the throat. Now and then these sensations extend up along the side of the neck towards the ear. An entire indisposition to talk is not unusual, for it requires an effort, or may excite cough. In almost all cases there is an everlasting disposition to heck and hem and clear the throat, present sometimes even in the sleep.

A gentleman applied to us in eighteen hundred and forty-three in the last stages of simple, uncomplicated throat-ail; he could swallow no food; even liquids returned by the nose; the pain was terrible. He starved to death.

*Croup* is so common a disease among children that it requires no description here; it affects the windpipe. As it attacks suddenly, most often in the night, and as an hour's time may be all the difference between life and death, it is proper to state the most reliable course to be pursued *until a physician can be obtained.*

1st. Keep the feet warm by having a jug of hot water kept against them; let them also be well wrapped up in woolen flannel.

2nd. Have a bucket of water almost as hot as the hand can bear. Have two pieces of woolen flannel of several thicknesses, one being on the throat while the other is in the hot water, renew every two or three minutes, until relief is given or the physician arrives. The water in the bucket must be kept hot by the constant addition of boiling water.

*Bronchitis* is a disease of the branches of the windpipe, which are the tubes, conveying the air from the windpipe to the lungs themselves. The distinguishing feature of Bronchitis, as above stated, is a stuffed up feeling. The eyes and nose water very much. There is a sensation of oppression. In fact, Bronchitis is a common cold, lasting for many days. But custom has given the name of "*Bronchitis*" to the symptoms of a common cold when they have become permanent. Properly speaking, this is *chronic bronchitis*, for shortness called "*bronchitis.*" The reader will do well to remember that "*bronchitis*," that puzzling name, that mysterious, that fondly hugged designation, pronounced so often, so glibly and familiarly by the deluded consumptive, is a common cold protracted. And as in a common cold the cough is not the first symptom, not appearing sometimes for a day or two, and then becomes the main feature: so the first symptoms of bronchitis are as above stated, but they end in a cough, which soon becomes the all-absorbing symptom, tearing and racking the lungs day and night, with scarcely any intermission sometimes, and in this, is strikingly different from consumption, for its cough is mainly at night and in the morning.

While, then, throat-ail is an affection of the voice-making organs, and croup is located in the windpipe, and bronchitis belongs to the air-tubes, which come out from the windpipe as the branches of a tree come out from its body, diverging widely; so consumption is a disease which attacks the lungs themselves, answering to the leaves of the tree, the lungs being at the extreme points of the air-tubes, as leaves are at the extremities of the branches of a tree.

But it is of consumption that these pages mainly speak. No doubt some degree of minuteness will be acceptable to the great mass of readers.

Imagine each leaf of a tree to be a small bladder or air-cell, filled with air, reaching them through the branches which draw their supply from what passes along the windpipe derived from without. These air-cells are of various sizes, from a pin head to a pea, and thinner than any paper we know of. All over these air-cells, like a vine on a wall, there are branches of bloodvessels, bringing the blood directly from the heart. These blood-vessels must necessarily be very minute, and to pass along them with any degree of facility the blood must necessarily be very pure, that is, it must not be thick, must not have in it foreign matter, sediments, the wastes of the system, its impurities. Mush will not readily flow through a hose-pipe. If water was so filled with mud as to have the consistency of stirabout, all the efforts of our gallant firemen would be in vain. This idea is of such vital importance, theoretically and practically, that the reader is earnestly desired, before he proceeds farther, to master it fully.

Of not less importance is it to remember a familiar fact that if a hose-pipe lays in a direct line, the water passes along with great ease and power, but if the hose are laid crooked and angular, even the purest water moves slowly.

If you take a common bladder, fully distended with air and draw straight lines from its neck to the bottom, those lines will become very crooked, if a great portion of the air be allowed to escape.

In health, the lungs are fully distended. The blood vessels are, comparatively speaking, in a direct line. The blood itself is pure, and from both causes it courses along the channels of life with rapidity and ease.

The first foundations of consumption are laid in the want of free breathing; the consequence, instantaneous and inevitable is, that the little blood-vessels stretching along a distended air-cell become tortuous, winding, doubling, thus retarding the flow of blood, and *retardation is death.* The moment the life-blood stagnates, that moment it begins to die, and in approaching actual stagnation it becomes corrupt, impure, thick. But more blood coming in from behind, the pressure becomes greater, the sides of the blood-vessels become distended and at last begin to yield, and there is an oozing through of the more liquid portions of the blood in the shape of distinct atoms or drops, which as they ooze, become hard, as the gum does from a puncture of the bark of some tree; this oozed blood-particle, hardened, is the hateful TUBERCLE, the seed of consumption and death.

An atom ever so small takes up room, and millions of them amount to a great deal, hence the room, in the air-cells, already diminished by the want of full breathing, and further by the detention of the blood in the tortuous blood-channels, is still farther taken up by the hard tubercles, so that from the three causes, there is very little room for any air at all; thus it is that *shortness of breath* is never absent in any case of consumption. In fact, it is an early symptom, and comes on by slow degrees, so slow as to be imperceptible; *it comes on months before any cough is noticed.*

But another result springs from the increased diminution of room in the lungs, caused by tubercles of all sizes from a white mustard seed upwards; they help to intercept the flow of blood along the veins and arteries, and the pressure from behind still continuing, the blood-vessels cannot bear the strain, and burst, pouring out the blood into the lungs, that is, bleeding of the lungs, spitting of blood, *the forerunner of death.*

Spitting blood is present in perhaps two-thirds of all who die of consumption.

When blood appears as a mere speck or drop or streak in the saliva, it is not a symptom worthy of notice. In any other form, it is the knell of death in men. In women, the mere spitting of blood, if during the periods, is no critical symptom; does not indicate the presence of tubercles necessarily. In men, it does. That is, when the blood is mixed up with the saliva, or comes clear, from half a teaspoonful at a time to a quart, tubercles are largely present, and in about two years the man will die, unless this symptom is removed.

Spitting of blood relieves the over fullness of the lungs, and diminishes cough remarkably, sometimes. Thus it is that bringing up a mouthful or two at a time, at intervals, is a relief, and may protract life for several years longer than would have been the case had it not been a symptom. Women losing blood naturally and periodically, thus protract the disease indefinitely.

We have recorded the birth of tubercles as founded in a want of sufficient distension of the air-cells by full breathing, to give the blood-tubes a direct line of conveyance.

But it is important to observe that precisely the same result will follow, if the blood becomes thick, mush-like. The blood-tubes may be ever so straight, yet if the blood be thick with impurities or from being of an imperfect material, the blood vessels will, if but moderately distended, allow the oozing through of its thinner particles, and give rise to tubercle. If the distention is intensified, then they burst their sides, and there is *Hæmorrhage of the Lungs.* In plain English, spitting of blood.

Thus we have come to two great important practical facts:

The want of full breathing gives birth to tubercle.

The want of pure blood gives birth to tubercle.

And here we have the two universal causes of Consumption:

IMPERFECT BREATHING. IMPURE BLOOD.

Surely it will not be difficult to remember these two things. We thus can plainly see how it is that persons who sit a great deal become consumptive; and any one may apply it to himself in the various occupations of life, without any further specifications as to this branch of the causes of Consumption.

More time will be spent in considering the other great branch of causes, *Impure Blood*, because it is not generally understood what are the more general causes of impure blood, and they ought to be generally known.

The heart has two suits of rooms, one filled with impure blood, going to the lungs to be purified; the other containing the purest blood of the body, which having undergone purification and perfection in the lungs, has been returned to this other side of the heart, to be propelled therefrom to the most distant portions of the human frame, imparting in its progress, renovation, restoration and life. The right side of the heart contains the impure, imperfect blood, while the pure blood is found in the left. But it cannot get from the right side into the left, without passing through an out-house, the Lungs, where the purifying process is carried on; and how?

We have seen that the blood is in the little branches of blood-vessels spread like a vine on the walls of the air-cells, the lungs, distended by air. Now, the blood does not come in actual contact with the air, the membrane of these minute vessels, thinner than the thinnest paper, manufactured only in Heaven, by omnipotent skill for the express purpose, is between the air and the blood. But a most wonderful process goes on here; there is a passage of substances through these membranes, the life of the air, the oxygen, as we say, passes out of the air-cell into the blood in the blood-vessels, and the impurities, the death of the blood passes from the blood-vessel into the air-cell, and in a moment the dead blood is made alive, and the air so pure from without but a moment before, is now deadly. Sc the death of the blood and the life of the air pass through these membranes, as light passes through glass or as electricity along the wires. Thus the Lungs are the great 'Change of life —the market place where Vitality and Death change their wares, the air being the nobler of the two, for while it takes death from the blood, it gives its own life therefor, the savior of physical humanity.

Let the most careless reader note and feel here, how impossible it is for the blood to be purified unless he breathes abundant pure air. The importance of breathing it constantly is strikingly exhibited in the established fact, that every ounce of blood of the whole body is thus aired every two and a half minutes of our existence. Thus the breathing of a pure air for so short a time as two and a half minutes imparts purification and refreshment to the whole human frame. This explains the instantaneousness with which persons are revived when taken into the air after confinement to a close room or crowded apartment for some time.

Thus it is, that after writing, or reading, or sewing, in one position for a long time, and the whole body feels tired, we get up, stretch the body, draw a full deep breath and walk across the room a few times, there is a feeling of rest and refreshment comes over us which is most agreeable. Why? Because the full breath distends the air-cells, straightens the blood vessels, the blood passes onward, presenting itself as it passes, to the life giving influences of the air in the freshly and fully distended air vessels. What madness it is, what deliberate suicide, to repress these yearnings of our instincts for the life-giving agencies which a beneficent Providence has thrown around us with such bounteous profusion: the Pure Air of Heaven!

But how does the blood become thus impure at the right side of the heart, before it goes for renovation to the lungs? There are two sources of impurity. A barrel of the purest water will be sadly defiled, if taken to the attic, and every floor in the house is washed with it, down to the cellar. The blood starts from the lungs pure and clean, it goes through the whole frame, washing out as it goes along, all the particles of our body which have died since the last visit; for we are always dying, reader! Particles which have subserved their uses, and having answered the great end of their creation, must be swept away as the cinders from the grate or the ashes from the hearth. Thus the blood, so pure but two and a half minutes before, is now loaded with offal, and is deposited in the heart, the great Clearing House of the body. So this body of ours is swept out, is washed clean every two minutes and a half of our existence. Like a magnificent steam engine requiring the constant attendance of the engineer, who if he does his duty, is all the time cleaning and oiling, so as to keep it in perfect working order, so is our body.

Does not the reader see, then, that not only is the want of full breathing a cause of impure blood, but that if the air he breathes is not pure when first breathed, it can no more unload the blood of its impurities as perfectly as it ought to have been done, than dirty water can wash a garment clean? You, who habitually breathe an impure, that is, confined air, for all confined air is impure, are a moral suicide. Hurry then, from your bed-chamber the instant of rising; hoist the windows of your sitting apartments, fling wide open your doors, divers times daily, even in the coldest weathers, and let out the death, instead of drawing it into your own system, to fester, and corrupt and rot you.

The other great cause of blood impurity at the right side of the heart, is the following:

We eat to live. What we eat is turned into blood, the object of that blood is two-fold. First, to keep us warm. Second, to repair the wastes of the system. Washing these wastes away in the manner we have named, is a matter of secondary importance, as to the blood; it is rather an incidental work. To keep us warm and to repair, these are First Things.

The process of converting food into blood is as follows:

After entering the stomach, it is converted into a sweetish whitish fluid in about two hours, when it is gradually passed out of the stomach along the intestines down to the vent of the system, receiving as it passes out of the stomach, drop by drop, the bile from the liver. In about four hours after eating an ordinary meal the stomach is empty, and in another hour or two we begin to get hungry. Opening into the stomach and all along the intestines, there are multitudes of open-mouthed tubes whose office it is to absorb, or withdraw what is real nutriment, from the passing mass of food, its essence. Some of it is ready to be withdrawn while in the stomach, other portions only become ready at various points along the intestinal passages, some only at the end. Thus it is that some elements of food are not converted into nutriment until long after having passed out of the stomach. These diminutive tubes convey their contents towards the great central tube of the system, just as the various springs, rivulets, creeks, &c., of any of our great rivers, flow together until all are united in one magnificent stream, which itself is finally emptied into the boundless sea. The heart is the great receiving sea of the myriads of nutriment-bearing channels of the human system. This nutrimental material enters the heart at the same time that another great river pours its contents into it; that river of blood which started from the heart a very few minutes before, and having washed out the body, delivers the defiled mass into the heart again to be renovated, refined, vivified. So that at any moment, the right side of the heart is industriously receiving two different kinds of fluid, the washings out of the body, and the imperfect nutrient material for blood, just as the Mississippi and Missouri pour very different waters together at their uniting point, soon mingling, however, into one homogeneous stream. The impure blood and the nutrient material soon coalesce, commingle and enter the lungs for purification, thoroughly mixed together. There meeting with the air, the nutrient fluid is in an instant converted into pure blood, and in the same instant of time, are the washings of the system converted into blood equally pure, by having had all its impurities abstracted at a breath. Thus we see, that in reality, our food does not become living, actual blood, until it has entered the lungs and been exposed to the life-giving influences of the air therein; hence we see that if air has not its life, that is its purity, it is utterly impossible for the food we have eaten to receive that finishing stroke which makes it real, perfect blood. And if not perfect, the system is imperfectly fed, and debility and disease are inevitable.

After the air in the lungs has given the finishing stroke, which makes pure and perfect blood out of the heterogeneous mass before described, it is sent back to the great receiving reservoir of the system, the left side of the heart, and is sent by thousands of *distributing* pipes, or blood vessels, to every fibre of the human frame, to be made into flesh, and bone, and joint, and ligament, wherever renovation is needed. And how minutely grand the process. The instant the air meets the impure and imperfect fluid mass in the lungs, it is converted into life, as instantaneous as chrystalization, as quick as the very lightning. This life consists in forming a little boat or cell, like a Nautilus on the sea; in this boat is an atom of life-giving life, which is freighted along the current of the blood, until it arrives at its destined port; the instant of its striking, the vessel is broken, the living atom, as instantaneously as the needle to the armature, bounds to its new home and is a part of the living man, in its turn to die and be washed away to make place for others. How wonderful is our life! How grandly mysterious, and how beautifully wise, is He who made it.

The reader has no doubt felt long ago in this narration how doubly essential to human health is the pure air of heaven, for it alone can purify the blood; it only can make blood out of the nutriment of the system. How infinitely essential, how gloriously useful is PURE AIR AND A PLENTY OF IT, in making the human frame all that it ought to be—all that it was intended to be.

But if the food be imperfect, its nutritive essence must be imperfect, and no air, however pure in quality, or in quantity large, can make a perfect blood out of it. We thus arrive at a sweeping general fact, that in order to have a perfect life-giving blood under the most favorable circumstances, the food we eat must be perfect.

The vegetables we cook must be fresh and perfect of their kind. The meats we consume must be the untainted meat of healthy animals. And both vegetables and meats should be properly and well cooked, and no more.

But to return to the new-born *tubercle.* How does it destroy the lungs? In going into an apple orchard, some trees appear to be well filled with fruit, equally distributed. Other trees have bunches of apples in patches, and by reason of varied exposure to the sun, we observe apples ripened in one spot, ripening in another, and quite green in a third. So it is, if we could see the lungs of people. In some, tubercles are thickly and equally distributed over the lungs. In others they are scattered about, a patch here, another there, a third yonder. A patch ripening in the first place; just beginning to turn in the second; while in the third, they are young and hard, and may never be different. A blackberry patch is a good and useful illustration of this point. It is the key which unlocks all the mysteries of quackery. There is a truth here, which every consumptive should understand, for there is more curative virtue in it, than in all medicine. It is wonderful how it has been lost sight of professionally. It is amazing how people won't see it. And the honest physician remains but a Cassandra still—a prophet, whose teachings, truth as they are, are wholly disregarded. But more of this in another place.

As an apple grows, it takes up more room, and soon touches its neighbor. Tubercles increase, meet, soften, and rot away together, eating up the lungs as they go. That is consumption. But what makes them grow, and what makes them soften and decay away together? The nascent crude tubercle may remain stationary for half a century; may be inappreciably hurtful; may and does remain innocuous for a life-time; may be as harmless to the system as powder is harmless, if fire is kept away. In proof of this a fact is stated—a fact of every-day occurrence in the dissecting-room. Out of fifty people, dead of other diseases than consumption, and being over forty years of age, scarcely one will be found who has not more or less tubercles in the lungs. This important fact is conclusive as to one interesting point: tubercles do not necessarily destroy life, as they may lay dormant for a life-time.

But what causes the tubercles to enlarge, soften, and rot the lungs away? Instead of writing down a long list of specifications, some of which might be omitted and many forgotten, it is of prime importance to notice one effect, instead of a hundred different causes.

Tubercles enlarge and are softened by debility of body, long protracted. Whatever then has a debilitating effect on the body, whether of a mental, moral, or physical nature, is the match which fires the magazine of life and burns it to ashes. Whatever keeps the body in a debilitated condition for weeks together, is capable of softening tubercles. If there be a great many tubercles of about the same age, as it were, any debilitating cause, acting for a comparatively short time, commences the decay, which, from the number of tubercles, soon becomes general, and the constitution fails rapidly. This is rapid consumption. It is like a spark applied to a wooden tenement which has been standing for half a century—every inch of wood is a tinder-box.

The evidence of softening tubercle is the spitting up of mouthfuls of yellow matter, which falls lumpily or heavily on the floor, with uneven edges, just as if one had been chewing a rag or piece of paper somewhat soft, and thrown it on the floor. If spit in water it sinks rapidly to the bottom, or if spit into a cup where there is but a spoonful or two of water, and the cup is tilted, the contents run rapidly from side to side. When an ulcer breaks or the lungs are decaying rapidly, the matter expectorated is not unlike thick rich cream.

A truth is about being stated, whose importance is such, that the whole civilized world should keep it in remembrance. As in a tree there may be a single cluster of apples, so in the lungs there may be but a single cluster of tubercles, and the remainder of the lungs may be perfectly sound. Or there may be two clusters or a dozen; each cluster may be a large or a small one; or they may be of various sizes. The symptoms of a ripening cluster are, first, a slight unfrequent cough; then more decided, still dry; next a little mucus comes; soon a large and free expectoration of yellowish matter is observed. If the cluster be large, this yellow matter is not brought away fast enough, and it is reabsorbed into the system. This reabsorption—this mingling of the matter of decayed lungs with the blood again—gives fever, hectic, night-sweats. As soon as the decayed matter of tubercle is removed, the patient begins to get better; the cough has disappeared in great part, if not wholly, the appetite improves, strength returns, flesh is gained, and the man may live half a century.

Whatever was done remedially at the time when the matter was about got rid of, gets the credit of having cured a man in the very last stages of consumption. The ignorant administrator and the more happy recipient, are willing enough to give the remedy the credit, and with all due formality a magistrate is sought, the declaration written, the hat pulled off, the bible procured, the hand held up, the head bowed, the deponent there affirming that

"I, John Lubberlie, was supposed to be in the last stage of consumption in the year 'forty-eight, suffering at the same time under a severe attack of rheumatism, liver complaint, dropsy, gravel, and cholera morbus. Simultaneously, also, I took the yellow fever, bilious colic, and small pox; the latter assuming the chronic form of scrofula, completely destroying my lungs, liver, spinal marrow, nervous system, and the entire contents of my phrenology. I finally got so low that I did not know my brother-in-law when he came to borrow money. For three months I swallowed nothing but twenty packages of Kunkelhausen's pills, which effected an immediate cure in three weeks.

"My uncle, Bacchus Pottinger, was afflicted so long with the gout that his life became a burden to him. He took only four boxes of said pills and life was a burden to him no longer. Further deponent saith not.

"Sworn and subscribed to, &c., &c."

Or if the patient happened at that critical time to go to the South, or North, or do any extraordinary thing, or any silly thing, such as drinking mule's milk, or goat's cream, or tar water, or brandy smash; if he had slept in a pig-pen, or cow-house, or inhaled hot water, or cold alcohol, or any thing else, the thing last done, has the credit of cure; and thus it is, that although the very next person who "tried" the same remedy died under it, the report has gone abroad, and like the cork leg, couldn't stop itself, and is going yet. Thus the world is full of cures, and any man you meet can deliver at sight half a dozen, any one of which cured a friend of his who was a great deal worse than you are. But to the crushing disappointment of multitudes, the experience is sadly uniform that "whatever it may have done for others, it has not availed for me."

If there be two or more patches of these tubercles, another softens, as the causes of softening are applied, and the same routine is gone through, perhaps until the dozenth time, which being the last, he may live on, to die many years after, of some totally different disease; or if the constitution be not strong, the man succumbs under these repeated attacks, and passes away.

The practical uses to be made of this narration of undoubted facts are various and important: first, it is useless to take any thing without the advice of a regular physician, who must be acquainted with every constituent of the remedy, so as to know in what direction its curative agencies tend; second, do nothing which common sense, joined with professional science, does not indicate as rational and wise.

The reason of these inferences is, the wide difference between an antecedence and subsequence and cause and effect. It is clearly irrational to adopt any remedy, simply because it was applied and restoration followed its application. The scientific practitioner takes no such grounds. It is not until after repeated experiments, made under every variety of circumstances, extending through months, and seasons, and years, giving a uniform result, that he lays hold of any remedy, but once laid hold of, he never rejects it for a single nor for a dozenth failure. Such is the difference between scientific medicine and quackery, between intellect and ignorance. It is only after many a long year's trial, that the skilful practitioner can be brought to say of any remedy, "I gave this, and it cured him." The charlatan speaks thus after the first trial, his ignorance sustaining his effrontery.

Hope is the highest remedy of the soul, the most efficient for the body. This Cluster Doctrine is a true groundwork for it, in consumptive disease. Surely it is Nature's remedy, for who among a thousand does not hope to the end, in consumption? The mischief lies in not making that hopefulness the ground of practical action. The consumptive hopes but does nothing, and thus it is that by hope he lives and dies.

Some of the best medical minds in the world, men who have spent a quarter of a century in examining the lungs of the dead, state to us this important, every day fact, that few people die, after forty, who have not in the lungs, the signs of having the consumption, without ever having had the slightest suspicion of the existence of the disease, and who finally died of maladies having no approximation towards it in nature. These signs, are scars of various lengths, little excavations, or cavities or puckerings of various sizes; all very small it is true, but still showing the great fact, that decay once existed there, and that the lungs may perfectly heal after having been divided or broken, or pierced, as numerous cases bear witness in the perfect recovery of men who have been stabbed in the breast, or shot through the lungs.

The great curative principle, to which the reader's attention is specially solicited, is this: In any attack of consumption, or its repetition, the patient should hope it was the last cluster to soften, and that if he can only weather this storm, it may be the last, and life and happiness may be his, for long years to come. Too much attention can scarcely be paid to this idea, and we hope every invalid reader will sleep on it nightly, and make it the ground of active, strenuous effort for health, every succeeding day, even until life's close, for the truly brave die striving. This being their motto, they do, in these diseases often, very often, outlive the prognostications of ignorance and presumption, for it is only such who can peril a prophecy of recovery or death; they speak firmly, where the physician gives opinions with trembling on the tongue.

Another practical fact, it is difficult to refrain from mentioning here:

On the partial or complete recovery from an attack of consumption, do not, as you value life, intermit a single possible effort for maintaining the highest possible degree of health; keep it up, until a habit of health is established, and even then, until the close of life, make it your study to live rationally, apportioning your eating to your exercise, as true wisdom dictates.

The symptoms of consumption have been described in a general manner. It is purposed under this head to speak of the far-off symptoms, which, if promptly treated, may eventuate in cure, with as much certainty as belongs to ordinary diseases. It is scarcely to be hoped that any attention will be paid to these, yet a book on this subject could not have claim to completeness of history without discoursing something on this head. It is certainly desirable, for it is highly practical, capable as it is, of making Consumption of the Lungs a manageable disease. But it is sadly feared, that it is to be the consummation of future centuries.

It is with consumption as it is with cholera, easily manageable in its first stages; in its last, utterly incurable. All men looked with horror on the Asiatic curse when it first visited our shores. When it was first described as sweeping the world with death, it was represented to be as instantaneous as a plague or palsy, and without one single warning note, hurrying multitudes to the grave; and yet on more minute and scientific inquiry, it is an established fact, that cholera, when attended to, in its premonitory stages, is an easily manageable disease, and is now shorn of half its horrors.

So of consumption, if we took note of its far off symptoms, and would then enter upon a course of life wisely energetic, it becomes one of the most manageable of diseases. The important practical inquiry then arises, what are the earliest and most invariable symptoms of common consumption of the lungs?

*Cough* is not an early symptom of consumption, necessarily, for there are many cases on record, in which cough was not an observed symptom, until within two or three weeks of death, and on examination, the lungs presented a diseased mass, burrowed with cavities.

*Spitting blood* is not an early symptom of consumption, necessarily, for about one-third of those who die of that disease, do not spit blood at all.

Among the very earliest symptoms of forming consumption, are combinations of the following; not all, perhaps, observable in any one case: A quicker pulse than common, a paler face, easily chilled after eating, more readily put out of breath than common; less fullness of flesh than usual at the corresponding season of the year, an unusual feeling of unrest on getting up in the morning, a greater tendency to coldness of the hands and feet, every now and then a day passing without any action of the bowels, with a very bad taste in the mouth when first waking up in the morning; a cold is easily taken, is more frequent, and lasts longer and longer, until one cold runs into another, making the confirmed cough, so ominous of approaching ill.

It will be seen at a single glance, from these symptoms, that they all indicate one thing, and that one thing is at the bottom of every case of consumption—*a want of vitality*; that is, a want of general vigor of system, of constitution.

But of all the things named, it will be more practical to select the two which are seldom, if ever absent, in any of the above combinations which result in consumption; hence it is import ant to be at some pains in stating them in their bearings. A quick pulse and a short breath pervade the disease from its earliest beginnings, during its entire progress, and down to its fatal end. Multitudes of lives might be saved yearly, if these two symptoms were promptly and wisely attended to. The importance of so doing, no language can adequately portray, and if it did, the people would not attend to it, with only here and there an exception. But a great truth is of small seed and of slow growth; yet that growth is certain, and its spread uncontrollable—the more so, as education becomes more general.

The PULSE beats about sixty-eight times in every minute of healthful adult life. The range is from sixty-six to seventy-two. When it is below sixty-six, there is something at fault; when it is over seventy-two, during all the hours of the twenty-four, there is always disease; and if it continues so for weeks and months, there is the strongest ground for apprehension that consumption is approaching.

There are intelligent men in the profession who will not coincide with this statement, but it will be because they have not had the opportunities of observation.

Whatever may be said of auscultation, of plessimetry, of sounding, of expectoration, there is in none of these a guide so sure as the condition of the pulse, with the aid of a competent interpreter; more, it is worth, to such an one, all the other modes of determination put together. It is said that the physicians among some of the Orientals are not allowed to see their female patients, the hand only being put out through the bed curtain, and by feeling the pulse, prescriptions must be made. If the powers of life are being pressed to death, the full, soft, slow pulse tells it in an instant; if active, and actual destruction of organic life is taking place in the body, the inflammatory pulse, quick, wiry, angry, spiteful, at once raises the note of alarm. Every physician knows how gratefully the pulsation, as of a woolen yarn beneath his finger, strikes upon his perceptions, on some urgent call, and how troubled if it gives the feeling of a quick vibrating small wire. The multitude of shades of difference between these carry with them their varied impressions, all highly instructive. In strongly-marked cases, however wan the patient may look, however hollow or fierce his cough, at first sight, an instantaneous feeling of the pulse is sufficient for the conclusion, "*You* have no consumption." But inasmuch as there have been cases of no appreciable activity of pulse, and even diminished pulse where consumption existed, the wise physician will never pronounce an opinion on any one single symptom. In some cases of spinal irritation, for example, there may be a troublesome hacking or hemming, and a quick pulse for months and years, without any special disease in the lungs. Still, this one broad fact should stand out prominently as an instructive beacon to all:

*A pulse steadily over eighty beats in a minute, for weeks together, is a forerunner of consumption.*

The physician in his kindness or hopefulness may tell you that some persons have a high pulse constitutionally, hereditarily, or some other plausible reason may be given for its presence in you; but if you are wise, with a pulse among the eighties, you will set it down as consumption begun, and will act accordingly.

ACCELERATION OF THE BREATHING is never absent in any case of actual consumption. In the last few weeks of life a few steps puts the patient out of breath, even if those steps be over a level floor. But long before this there was observed an inability to walk fast without considerable discomfort. In fact, a slow and measured tread is the symptom which first strikes the ordinary observer. The man himself may be scarcely an apparent invalid, except on close scrutiny. He may be lively in conversation, he may eat heartily, may have little or no cough, but any effort on your part to induce him to greater bodily activity is instinctively avoided. At a still earlier period one thing has been forced upon the attention of the patient: that he does not mount a pair of stairs with the same celerity as formerly. In days long ago he could take two or three steps at a stride, and even feel the better for it when he reached the top; but now, such an effort would make him puff and blow inconveniently. At an earlier period still, there is an observable feeling of tiredness about the legs and knees on going up stairs, a feeling of weakness there, not known in earlier years, implying a want of bodily vigor not pertinent to that stage of life.

It is to be hoped that no one will haste away with the impression that a little feeling of fatigue in going up a pair of stairs is a sign of consumption. This book is not written for quibbling critics; it is written for the instruction of people of sober views, who can look at a subject steadily, willing to be informed, but unwilling to run away with either end of the subject, or precipitate themselves into the weakness of extremes.

But it is an instructive fact, that if this easiness of fatigue in ascents, be conjoined with the quick pulse, and be so for months in succession, it is an impressive warning of coming consumption, and millions would be saved, if it were heeded.

A man may be lazy, it may be summer time, or various other things may give rise to a transient exhibition of acceleration in pulse and breath, or they may arise from the mere habit of sedentariness; but there is one easy, decisive, infallable method of determining whether these symptoms are from transient causes, or from an actual change going on in the structure of the lungs themselves; and that is, by measuring the quantity of air which the lungs are capable of drawing in, at one deep, full, free breath, that is done by the use of an instrument often seen in the street, "The Lung Measurer," or "Spirometer," as Mr. Hutchinson, of London, its inventor, names it. The first instrument of the kind ever made in the United States, was made for the Author of these pages, in 1847, since which time a number of eminent English practitioners have learned to employ them, and some few in this country. As a general thing, it has not found favor here, as it is expensive, is liable to abuse, and to the mass of physicians, the opportunities of making varied observations upon it, are not offered. Besides, it requires time and patience to classify the phenomena which it presents; and unless a man have a considerable practice of that kind, it does not pay, either in money or in data for scientific results.

In the Author's practice, then, the great preponderating indications of consumption are, accelerated pulse and breathing; no judicious practitioner will rely wholly on the pulse, or any other two or three symptoms, but on the whole set of symptoms which any given case presents, together with the history of his life, his temperament, his habits, his hereditary tendencies and idiosyncracies, that is, peculiarities, of constitution.

The more obvious symptoms of consumption have been already sketched in a general way. So few persons recover from what is called confirmed consumption, that it was not considered profitable to enter into a critical enunciation and description of all the symptoms, real or imaginary, and in their various stages, degrees and progress; such a thing would materially detract from the practical, utilitarian design, which has been ever prominent. There is so little hope of clearing out the Augean stable of a confirmed consumptive, in any given case, that it is considered only worth while to direct attention critically to the symptoms and stages which admit of a comparatively speedy and permanent arrest or cure.

The large majority of deaths by consumption, are out of married life, indicating the general fact, that its victims are mainly the young, from twenty to thirty. As dying at twenty, or soon after, proves the actual existence of the disease in its forming stages, while yet in the teens, our hope lies in parental influence and intelligence, for then, they can enforce by authority, that course of life, most appropriate towards arresting and removing the disease.

